# Sports activities at a young age decrease hypertension risk—The J‐Fit ^+^ study

**DOI:** 10.14814/phy2.15364

**Published:** 2022-06-27

**Authors:** Hiroshi Kumagai, Eri Miyamoto‐Mikami, Yuki Someya, Tetsuhiro Kidokoro, Brendan Miller, Michi Emma Kumagai, Masaki Yoshioka, Youngju Choi, Kaname Tagawa, Seiji Maeda, Yoshimitsu Kohmura, Koya Suzuki, Shuichi Machida, Hisashi Naito, Noriyuki Fuku

**Affiliations:** ^1^ Graduate School of Health and Sports Science Juntendo University Chiba Japan; ^2^ The Leonard Davis School of Gerontology University of Southern California, California Los Angeles California USA; ^3^ Faculty of Sport Science Nippon Sport Science University Tokyo Japan; ^4^ Department of Psychiatry David Geffen School of Medicine, University of California Los Angeles California USA; ^5^ Graduate School of Comprehensive Human Sciences University of Tsukuba Tsukuba Japan; ^6^ Japan Society for the Promotion of Science Tokyo Japan; ^7^ Institute of Sports & Arts Convergence Inha University Incheon South Korea; ^8^ Faculty of Sport Sciences Waseda University Saitama Japan

**Keywords:** blood pressure, DNA methylation, exercise experience, former athletes, young athletes

## Abstract

This study aimed to assess (1) blood pressure between young, current athletes, and non‐athletes early in life; (2) hypertension prevalence between former athletes and the general population later in life; and (3) understand the mechanisms between exercise training and hypertension risks in the form of DNA methylation. Study 1: A total of 354 young male participants, including current athletes, underwent blood pressure assessment. Study 2: The prevalence of hypertension in 1269 male former athletes was compared with that in the Japanese general population. Current and former athletes were divided into three groups: endurance‐, mixed‐, and sprint/power‐group. Study 3: We analyzed the effect of aerobic‐ or resistance‐training on DNA methylation patterns using publicly available datasets to explore the possible underlying mechanisms. In young, current athletes, the mixed‐ and sprint/power‐group exhibited higher systolic blood pressure, and all groups exhibited higher pulse pressure than non‐athletes. In contrast, the prevalence of hypertension in former athletes was significantly lower in all groups than in the general population. Compared to endurance‐group (reference), adjusted‐hazard ratios for the incidence of hypertension among mixed‐ and sprint/power‐group were 1.24 (0.87–1.84) and 1.50 (1.04–2.23), respectively. Moreover, aerobic‐ and resistance‐training commonly modified over 3000 DNA methylation sites in skeletal muscle, and these were suggested to be associated with cardiovascular function‐related pathways. These findings suggest that the high blood pressure induced by exercise training at a young age does not influence the development of future hypertension. Furthermore, previous exercise training experiences at a young age could decrease the risk of future hypertension.

## Graphical abstract

High blood pressure induced by exercise training at a young age is an adaptation to exercise training and does not influence the development of future hypertension. Furthermore, previous exercise training experiences at a young age could decrease the risk of future hypertension.

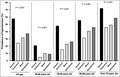

## 1 INTRODUCTION

High physical fitness levels and participation in sports protect against all‐cause and cardiovascular disease (CVD) mortalities (Blair et al., 1989; Kokkinos et al., 2008; Leong et al., 2015; Oja et al., 2017; Ortega et al., 2012). Recent studies suggest that former top‐level athletes have increased lifespans compared to the general population (Antero et al., 2021; Antero‐Jacquemin et al., 2018; Lemez & Baker, 2015; Takeuchi et al., 2019). For example, athletes who participated in the Olympic games had lower mortality than the general population (Antero et al., 2021; Antero‐Jacquemin et al., 2018; Takeuchi et al., 2019), and a systematic review concluded that participation in elite sports leads to longer lives (Lemez & Baker, 2015) in part by lowering CVD risk (Antero et al., 2021).

The effects of exercise training on cardiovascular function are different among sports types. Exercise is largely classified into aerobic and resistance exercise. Aerobic exercise, such as walking or jogging increases arterial compliance (Matsubara et al., 2014; Tanahashi et al., 2014; Tanaka et al., 2000), and a recent meta‐analysis demonstrated that the blood pressure‐lowering effect of aerobic exercise was similar to that of antihypertensive medications in people with hypertension (Naci et al., 2018). In contrast, resistance exercise using heavy weights decreases arterial compliance (Miyachi et al., 2004; Tagawa et al., 2018). Indeed, endurance‐trained, and resistance‐trained subjects showed higher and lower arterial compliance than age‐matched control subjects, respectively (Miyachi et al., 2003; Otsuki et al., 2007). Interestingly, it has been reported that detraining after the exercise training intervention completely returns the adapted cardiovascular functions to the baseline levels (Miyachi et al., 2004; Mustata et al., 2004). However, the association between previous exercise training experience and future CVD risk has not been clarified yet.

Experience of exercise training at a young age may remain in the body in some manner, which may influence CVD mortality and lifespan in former athletes. One possible mediator of this is epigenetic modifications, such as DNA methylation or histone modification. Epigenetic modifications are acquired alternations that are not caused by changes in the DNA sequence and regulate gene expression levels. Exercise training influences DNA methylation patterns (Voisin et al., 2015), and recent epigenome‐wide association studies (EWAS) suggested that both aerobic exercise training and resistance exercise training altered DNA methylation status after exercise training interventions (Lindholm et al., 2014; Seaborne et al., 2018). In contrast, DNA methylation patterns are different between healthy people and people with diseases such as obesity (Wahl et al., 2017), type 2 diabetes mellitus (Michael et al., 2015), and hypertension (Kazmi et al., 2020). Therefore, it is possible that exercise training influences the development of CVD risk factors by modifying DNA methylation patterns. Nevertheless, these possible associations have not yet been clarified.

Our study first compared blood pressure levels between young, current athletes and non‐athletes. Second, we compared the prevalence of hypertension between former athletes and the general population. Third, we explored the underlying mechanism by focusing on DNA methylation. Through these three studies, we aimed to understand the influences of current and past exercise training on blood pressure and explore its underlying mechanisms.

## 2 METHODS

### 2.1 Study design

The present study consisted of three individual studies. In the first study, we assessed blood pressure levels in young current athletes and non‐athlete controls to compare blood pressure between endurance, mixed, sprint/power athletes, and non‐athlete controls (Study 1). The second study assessed the prevalence of hypertension in former athletes and compared it to that in the general population using age‐sex matched national data (Study 2). Additionally, the hazard ratio of hypertension was calculated in the former athletes. The third study analyzed publicly available DNA methylation data before and after the aerobic and resistance exercise training interventions to explore the possible mechanism of the association between exercise training experience and hypertension (Study 3).

### 2.2 Study 1: Blood pressure levels in young participants

Young college athletes (*n* = 311) and controls with no regular exercise habits (*n* = 42) participated in the study. Measurements were obtained in a quiet temperature‐controlled room (24–26°C) after the participants fasted for over 3 h. The athletes were encouraged not to perform intense training 12–24 h before the measurements. A digital scale was used to measure the bodyweight of the participants to the nearest 0.1 kg. A wall‐mounted stadiometer was used to measure their height to the nearest 0.1 cm. Body mass index (BMI) was calculated by dividing the weight (kg) of the participants by their height squared (m^2^). After a resting period of at least 20 min, supine blood pressure and heart rate were measured two to three times with around 5 min intervals using a previously described noninvasive vascular profiling system (form PWV/ABI; Colin Medical Technology; Kumagai et al., 2018). Pulse pressure (PP) was calculated as systolic blood pressure (SBP) minus diastolic blood pressure (DBP). To compare the effect of the exercise type on blood pressure, athletes were divided into three groups: endurance (mid‐long running, *n* = 61), mixed (soccer, basketball, handball, volleyball, martial arts, tennis, and others, *n* = 212), and sprint/power (sprinting and throwing, *n* = 38) groups based on the sports club they belonged to. This classification is based on acute physiological responses (i.e., heart rate and blood pressure) and the long‐term impact on cardiac output and remodeling (Pelliccia et al., 2016; Pelliccia et al., 2017). Written informed consent was obtained from each participant in accordance with the tenets of the Declaration of Helsinki. The study was approved by the Ethics Committees of Juntendo University and the University of Tsukuba.

### 2.3 Study 2: Prevalence of hypertension in former athletes

The J‐Fit^+^ Study is a historical cohort study that included the alumni of the Department of Physical Education at a sports university in Japan (Figure S1: https://figshare.com/s/a313d23e0f709852a6e2). A total of 12,409 students graduated from the university between 1956 and 2018. After excluding the alumni who had no address information or died (*n* = 2902), we mailed self‐administered questionnaires to 9507 alumni in 2018. Of these, 2141 alumni (1794 men and 347 women) completed self‐administered questionnaires about their present physical characteristics, daily physical activity levels, lifestyle, history of sports activity, and medical backgrounds (diagnosis of diseases by a medical doctor, age of onset, and medication status), with a response rate of 22.5%. Alumni who use antihypertensive medication prescribed by a medical doctor were defined as hypertension. Current physical activity was assessed using the International Physical Activity Questionnaire, and alumni who underwent moderate or vigorous physical activity at least once a week were defined as physically active. Obesity was defined as a BMI ≥25 kg/m^2^ according to the Japan Society for the Study of Obesity. Smokers and drinkers were defined based on their current smoking and drinking status. Female alumni (*n* = 256) were excluded for the following reasons. First, because the Department of Physical Education at this university had not enrolled female students until 1991, the female alumni were relatively young (average age: 34 years old) to assess the prevalence of hypertension. Additionally, although CVD risk in females increases after menopause, most of the female alumni were under 50 years old (98.4%). Second, the sample size was not enough to analyze with stratification. Additionally, male alumni who did not provide an answer regarding a medical history of hypertension (*n* = 93), those with a lack of information about sports activities or non‐players during university days (*n* = 234), and those who were under 40 years of age were also excluded from the analyses (Figure S1: https://figshare.com/s/a313d23e0f709852a6e2). A total of 1269 male former athletes were finally analyzed. Similar to Study 1, the former athletes were divided into three groups: endurance (mid‐long running, triathlon, and others, *n* = 139), mixed (soccer, basketball, handball, volleyball, martial arts, tennis, and others, *n* = 659), and sprint/power (sprinting, throwing, jumping, gymnastics, baseball, and others, *n* = 471) groups. To compare the prevalence of hypertension between the alumni of the sports university and the general population in Japan, we used cross‐sectional data from the 2018 National Health and Nutrition Survey conducted by the Ministry of Health, Labour and Welfare as the data on the prevalence of hypertension in the general population. Same to the alumni study, hypertension was defined by the medication status. Written informed consent was obtained from each participant in accordance with the tenets of the Declaration of Helsinki. The study was approved by the Ethics Committees of Juntendo University.

### 2.4 Study 3: Effects of exercise training on the DNA methylation patterns

We used external EWAS datasets from the Gene Expression Omnibus (GEO) to examine the effects of both aerobic and resistance trainings on DNA methylation. The analyzed EWAS datasets were GSE60655 for aerobic exercise training using the Infinium HumanMethylation450 BeadChip array and GSE114763 for resistance training using the Infinium MethylationEPIC BeadChip array, published by Lindholm et al. (2014) and Seaborne et al. (2018), respectively. Because the Infinium MethylationEPIC BeadChip array includes >90% of the CpG sites from the Infinium HumanMethylation450 BeadChip array (Solomon et al., 2018), we analyzed the CpG sites based on the Infinium HumanMethylation450 BeadChip array. Skeletal muscle samples from seven and eight male subjects were obtained before and after 12 weeks of aerobic and 7 weeks of resistance exercise training, respectively. The raw intensity files (IDAT) of each dataset were imported from the GEO database into the R programming environment (v4.0.3) using R Studio (v1.4.1103). A differential methylation analysis pipeline, with quality control, filtering, normalization, data exploration, and statistical testing for probe‐wise differential methylation, was performed using multiple R Bioconductor packages including lumi (Du et al., 2008), limma (Ritchie et al., 2015), and missMethyl (Phipson et al., 2016). Unadjusted *p*‐value significance (*p* < 0.05) was used to create lists of differentially methylated CpG sites in each aerobic and resistance training dataset, and commonly assessed hypo‐ and hypermethylated CpG sites in both datasets.

### 2.5 Statistical analysis

All data are expressed as mean ± standard deviation or frequency counts (for categorical data). The Shapiro–Wilk test was used to assess the normality of all parameters. One‐way analysis of variance and the Tukey–Kramer test or the Steel–Dwass test were applied for continuous variables, while the chi‐square test was applied for nominal variables. Independent correlates of blood pressure were examined by performing multiple linear regression analysis. The association between the exercise type while attending college and the incidence of hypertension was assessed using Cox proportional hazards models. Data were adjusted for age, BMI, current physical activity, smoking status, and drinking status. Multivariable‐adjusted hazard ratios for hypertension and 95% confidence intervals (95% CI) were obtained using the former endurance athlete group as the reference. Statistical significance was set at *p* < 0.05. Statistical analyses were performed using JMP Pro version 12 (SAS Institute) and R programming environment (v4.0.3) using R Studio (v1.4.1103).

## 3 RESULTS

### 3.1 Blood pressure levels in young participants (study 1)

**FIGURE 1:**
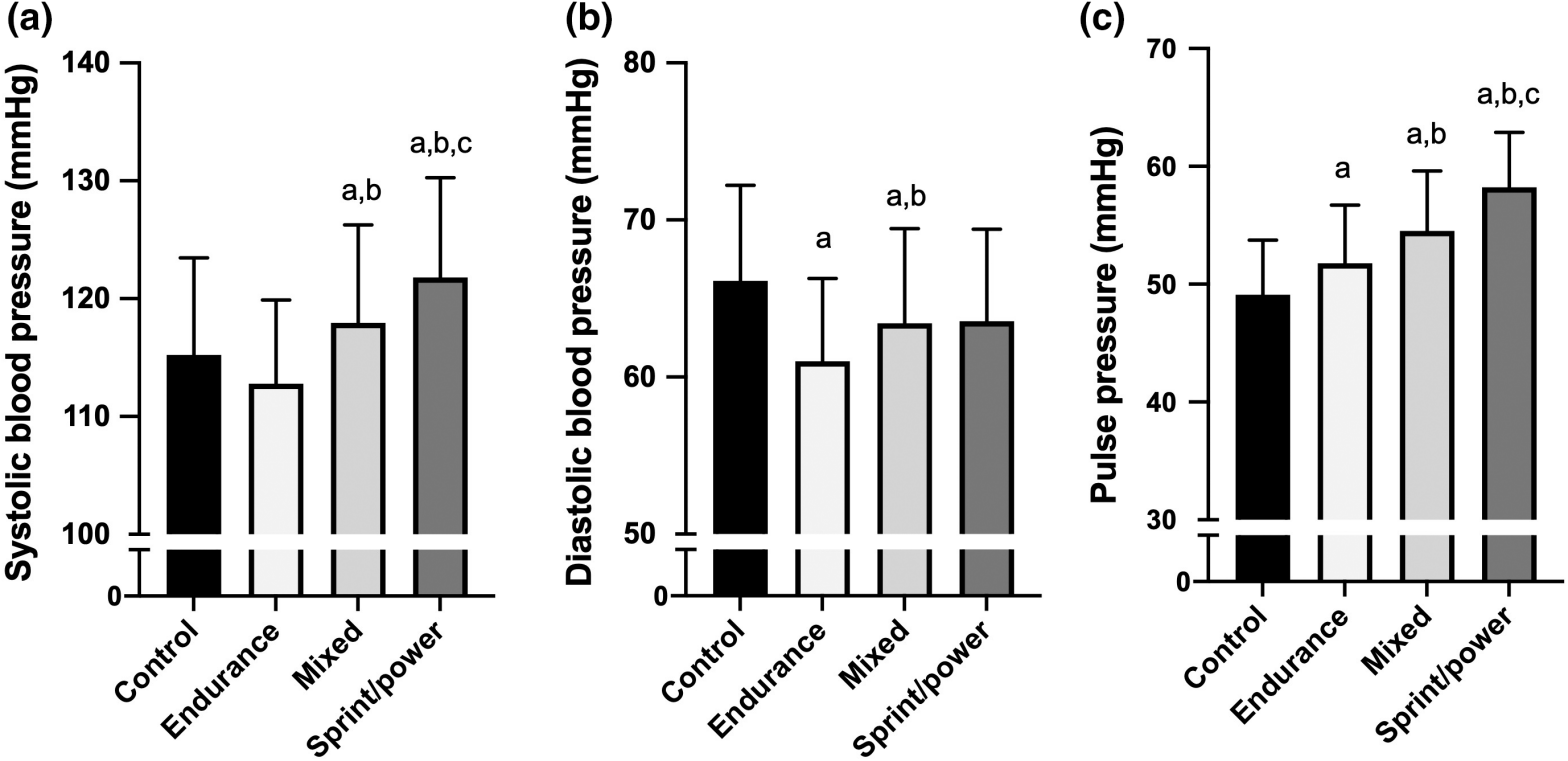
Systolic blood pressure (a), diastolic blood pressure (b), and pulse pressure (c) in current young athletes. ^a^ *p* < 0.05 versus control group. ^b^ *p* < 0.05 versus endurance group. ^c^ *p* < 0.05 versus mixed group.

The characteristics of young participants are shown in Table 1. The participants in the control group were significantly older than those in the other groups; 22.5 ± 1.5, 19.7 ± 1.4, 19.7 ± 1.3, and 19.6 ± 1.1 years old in control, endurance, mixed, and sprint/power groups, respectively. Height, weight, BMI, and heart rate were significantly different among the groups. Young athletes undergoing mixed and sprint/power training exhibited higher SBP, young athletes undergoing endurance and mixed training exhibited lower DBP, and all types of young athletes exhibited higher PP than non‐athlete controls (Figure 1). These significant associations remained significant after considering BMI.

**TABLE 1:** Characteristics of young subjects

	Control	Endurance	Mixed	Sprint/power	*p* value (ANOVA)
	*n* = 42	*n* = 61	*n* = 213	*n* = 38	–
Age, years	22.5 ± 1.5	19.7 ± 1.4^a^	19.7 ± 1.3^a^	19.6 ± 1.1^a^	<0.001
Height, cm	171.5 ± 5.7	171.8 ± 5.4	175.4 ± 6.4^a,b^	176.6 ± 5.6^a,b^	<0.001
Weight, kg	66.9 ± 11.5	58.4 ± 4.5^a^	69.5 ± 7.9^b^	81.6 ± 18.0^a,b,c^	<0.001
BMI, kg/m^2^	22.8 ± 3.9	19.8 ± 1.1^a^	22.6 ± 1.9^b^	26.0 ± 4.7^a,b,c^	<0.001
Heart rate, bpm	55.5 ± 9.2	50.4 ± 6.5^a^	54.5 ± 8.3^b^	58.4 ± 8.5^b^ ^,^ ^c^	<0.001

*Note*: Data are shown as the mean ± SD.

^a^ *p* < 0.01 versus control group.

^b^ *p* < 0.01 versus endurance athletes.

^c^ *p* < 0.01 versus mixed athletes.

### 3.2 Prevalence of hypertension in former athletes (study 2)

**FIGURE 2:**
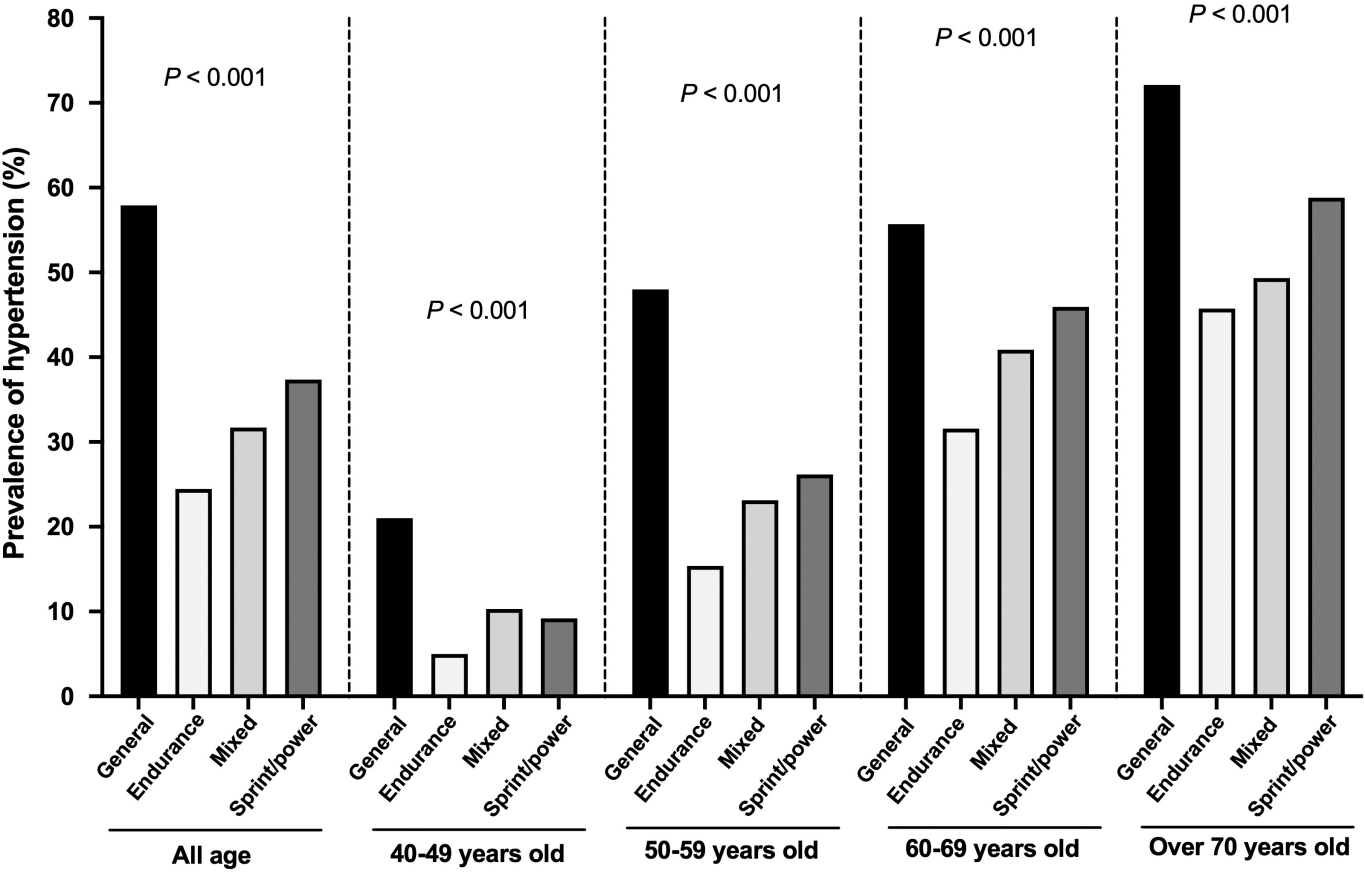
The prevalence of hypertension in former athletes and the general population. The percentage of hypertension in the general population was obtained from the 2018 National Health and Nutrition Survey conducted by the Ministry of Health, Labour and Welfare. People who use antihypertensive medication were defined as hypertension in both former athletes and the general population.

**FIGURE 3:**
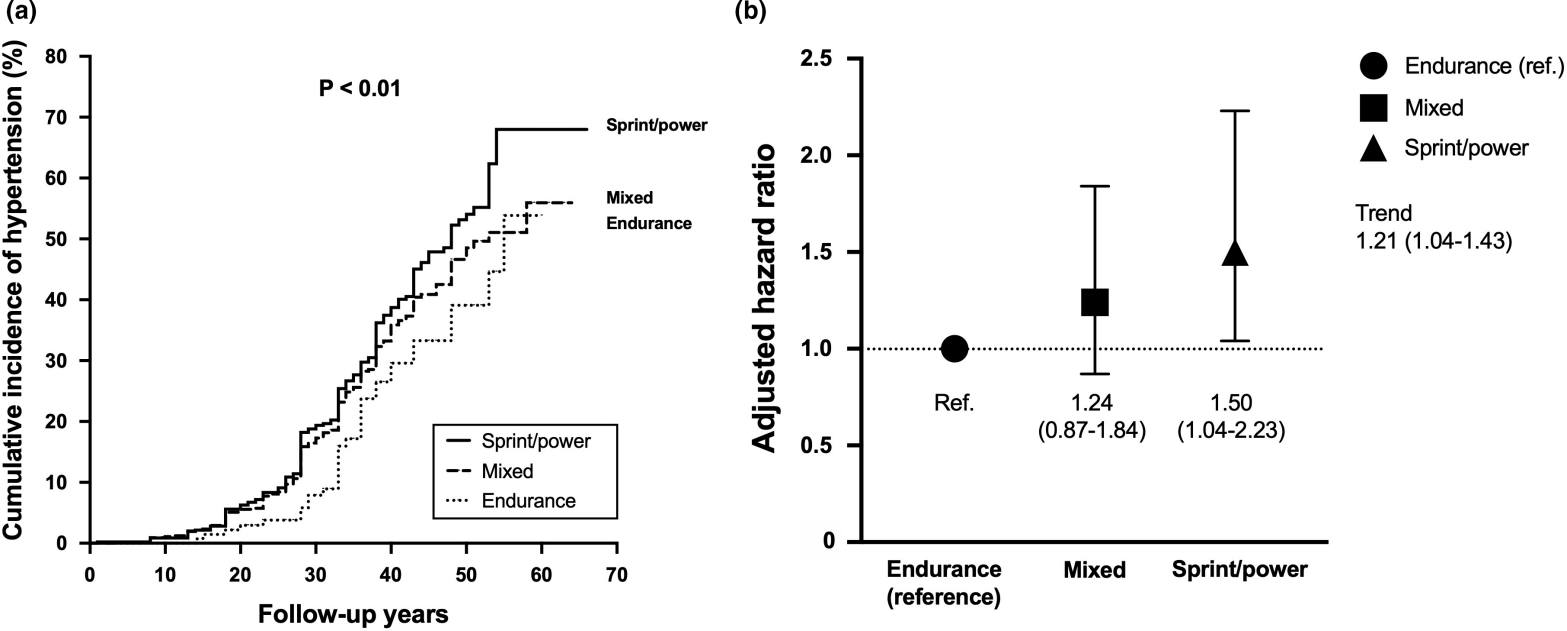
The influence of exercise training type on the risk of hypertension in former athletes. (a) Cumulative incidence curve for hypertension during the follow‐up period in former athletes. (b) Adjusted hazard ratios for the incidence of hypertension among former athletes. Values are adjusted hazard ratios (95% CI) to the former endurance athletes as a reference group and are adjusted by age, BMI, physical activity, smoking, drinking. Former athletes who use antihypertensive medication were defined as hypertension.

The characteristics of former athletes underwent endurance (*n* = 139), mixed (*n* = 659), and sprint/power (*n* = 471) training are shown in Table 2. Former athletes with current physical activity showed a significantly lower prevalence of hypertension than those without current physical activity (28.2% vs. 39.6%, *p* < 0.001). The prevalence of hypertension was significantly lower in the former athletes than that in the general Japanese population in all age groups (Figure 2). Furthermore, the former athletes without current physical activity also exhibited a significantly lower prevalence of hypertension than the general population in all age groups (Figure S2: https://figshare.com/s/a313d23e0f709852a6e2). The mean follow‐up periods were 37.6 ± 11.8 years, 38.3 ± 11.0 years, and 39.3 ± 11.3 years in the former athletes underwent endurance, mixed, and sprint/power training, respectively. Among the former athletes, athletes underwent endurance training showed a lower cumulative incidence of hypertension during the follow‐up period (Figure 3a). Although not statistically significant, similar trends were observed after considering the current physical activity (Figure S3: https://figshare.com/s/a313d23e0f709852a6e2). Unadjusted hazard ratios for the incidence of hypertension among former athletes were 1.00 (reference) in endurance, 1.37 (0.96–2.03) in mixed, and 1.62 (1.12–2.40) in sprint/power training groups. These associations persisted after adjustment for age, BMI, physical activity, smoking status, and drinking status: 1.00 (reference), 1.24 (0.87–1.84), and 1.50 (1.04–2.23) in athletes underwent endurance, mixed, and sprint/power training, respectively (Figure 3b; Table S1: https://figshare.com/s/a313d23e0f709852a6e2).

**TABLE 2:** Detailed characteristics of former athletes

	All age‐group	40–49 years old	50–59 years old	60–69 years old	Over 70 years old
Endurance	*n* = 139	*n* = 40	*n* = 26	*n* = 38	*n* = 35
Age, year	59.6 ± 11.8	44.8 ± 2.6	54.8 ± 2.5	64.8 ± 2.8	74.4 ± 3.4
Height, cm	169.6 ± 5.2	172.5 ± 4.4	171.2 ± 5.1	168.3 ± 4.5	166.2 ± 4.4
Weight, kg	66.6 ± 7.6	66.5 ± 6.8	67.2 ± 7.9	65.8 ± 8.1	67.0 ± 7.8
BMI, kg/m^2^	23.2 ± 2.5	22.3 ± 1.9	22.8 ± 2.2	23.2 ± 2.7	24.2 ± 2.6
Obesity, *n* (%)^a^	29 (20.9)	5 (12.5)	3 (11.5)	9 (23.7)	12 (34.3)
Physical activity, *n* (%)	91 (65.5)	28 (70.0)	17 (65.4)	28 (73.7)	18 (51.4)
Smoking, *n* (%)	10 (7.3)	3 (7.5)	0 (0)	5 (13.9)	2 (5.7)
Drinking, *n* (%)	111 (80.4)	32 (80.0)	24 (92.3)	30 (81.1)	25 (71.4)
Mixed	*n* = 659	*n* = 136	*n* = 173	*n* = 208	*n* = 142
Age, year	60.3 ± 11.0	45.3 ± 3.0	54.6 ± 2.8	64.3 ± 2.9	75.5 ± 4.8
Height, cm	171.5 ± 6.6	173.7 ± 6.8	173.0 ± 6.0	171.7 ± 5.6	167.3 ± 6.5
Weight, kg	71.4 ± 9.6	73.5 ± 9.9	72.8 ± 8.3	72.0 ± 9.7	66.7 ± 8.9
BMI, kg/m^2^	24.2 ± 2.7	24.3 ± 3.0	24.3 ± 2.3	24.4 ± 2.8	23.8 ± 2.5
Obesity, *n* (%)^a^	223 (33.9)	47 (34.8)	57 (32.9)	74 (35.7)	45 (31.7)
Physical activity, *n* (%)	387 (58.9)	95 (69.9)	108 (62.4)	119 (57.2)	65 (46.4)
Smoking, *n* (%)	117 (17.8)	31 (22.8)	29 (16.8)	38 (18.4)	19 (13.4)
Drinking, *n* (%)	521 (79.1)	112 (82.4)	150 (86.7)	174 (83.7)	85 (59.9)
Sprint/power	*n* = 471	*n* = 98	*n* = 107	*n* = 135	*n* = 131
Age, year	61.3 ± 11.3	45.0 ± 2.8	55.1 ± 2.8	64.7 ± 2.8	74.9 ± 4.0
Height, cm	170.9 ± 6.8	173.5 ± 6.0	172.2 ± 6.1	171.5 ± 6.6	167.4 ± 6.6
Weight, kg	70.5 ± 10.5	74.1 ± 11.1	71.8 ± 8.6	72.2 ± 9.9	65.2 ± 10.0
BMI, kg/m^2^	24.1 ± 2.7	24.6 ± 3.1	24.2 ± 2.2	24.5 ± 2.6	23.2 ± 2.8
Obesity, *n* (%)^a^	166 (35.2)	39 (39.8)	43 (40.2)	52 (38.5)	32 (24.4)
Physical activity, *n* (%)	263 (56.0)	66 (67.3)	62 (57.9)	73 (54.5)	62 (47.3)
Smoking, *n* (%)	77 (16.5)	21 (21.4)	16 (15.0)	23 (17.3)	17 (13.1)
Drinking, *n* (%)	380 (81.2)	85 (86.7)	93 (86.9)	111 (82.8)	91 (70.5)

*Note*: Data are shown as the mean ± SD.

^a^ Obesity is defined body mass index ≥25 kg/m^2^.

### 3.3 Effects of exercise training on the DNA methylation patterns (study 3)

**FIGURE 4:**
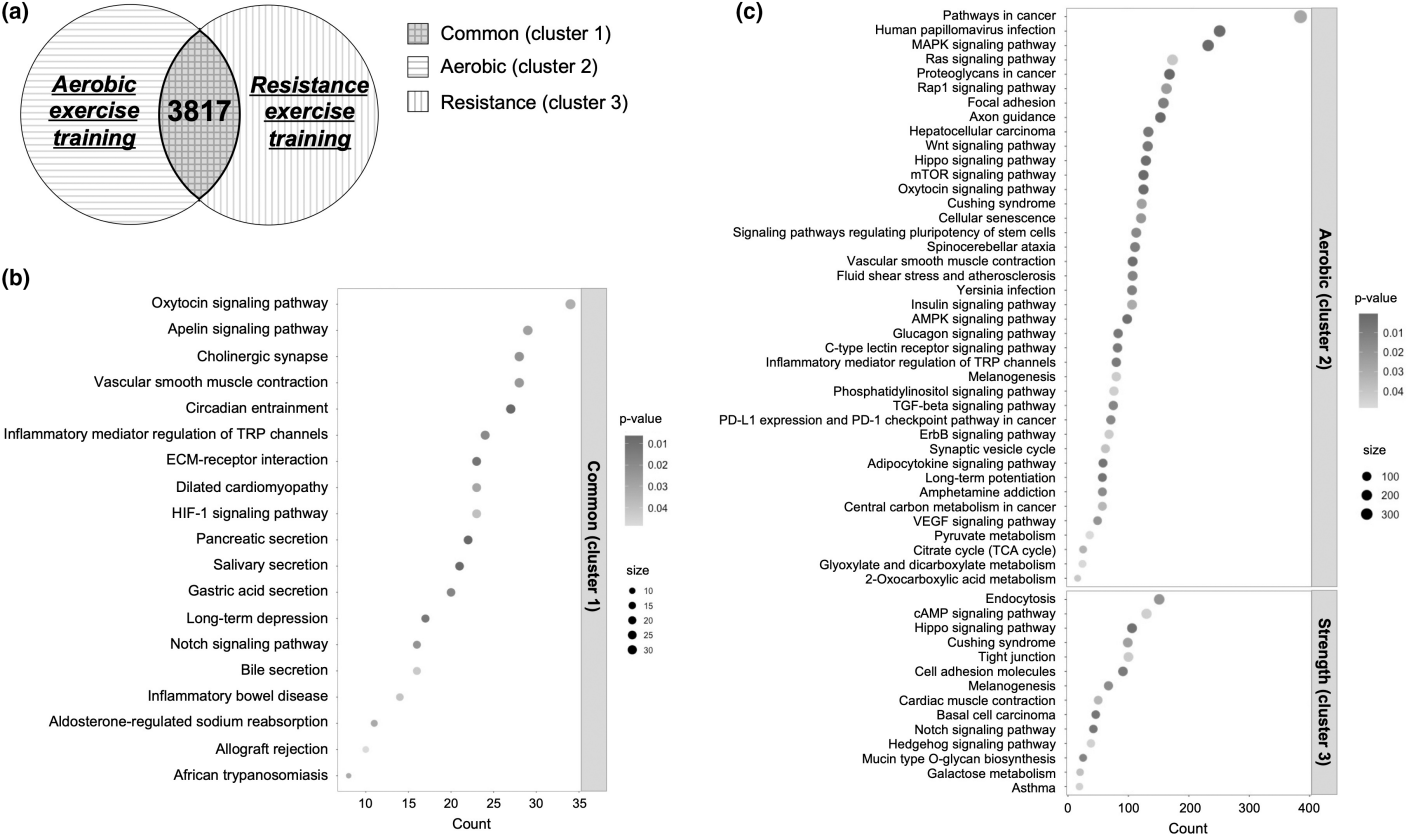
The effect of exercise training on the DNA methylation sites. (a) The number of CpG sites modified by aerobic and/or resistance exercise training. Cluster 1 is commonly modified by aerobic and resistance exercise training. Clusters 2 and 3 are CpG sites modified by only aerobic or resistance exercise training (Cluster 1 is removed from Clusters 2 and 3). Unadjusted *p*‐value significance (*p* < 0.05) was used to create the clusters. (b, c) Dot plots for the significantly enriched terms in the KEGG (Kyoto Encyclopedia of Genes and Genomes) enrichment analysis using cluster 1 (b), Cluster 2, and Cluster 3 (c).

Because both former athletes who underwent endurance and those who underwent resistance training exhibited a lower prevalence of hypertension than that in the general population, we explored the common mechanism underlying this observation by focusing on DNA methylation. Analysis using publicly available datasets showed that 3817 CpG sites were commonly modified following both aerobic and resistance exercise training (*p* < 0.05; Figure 4a). Significant enriched KEGG (Kyoto Encyclopedia of Genes and Genomes) pathways of Cluster 1 (both aerobic and resistance exercise training), Cluster 2 (only aerobic exercise training), or Cluster 3 (only resistance exericse training) are shown in Figure 4b,c. The KEGG pathways‐related to cardiovascular function, such as vascular smooth muscle contraction, apelin signaling pathway, cholinergic synapse, oxytocin signaling pathway, inflammatory mediator regulation of TRP channels, HIF‐1 signaling pathway, Notch signaling pathway, and aldosterone‐regulated sodium reabsorption, were significantly enriched in the Cluster 1 (Figure 4b). The CpG sites in Cluster 2 were also associated with cardiovascular function‐related pathways, such as vascular smooth muscle contraction, MAPK signaling pathway, Ras signaling pathway, Wnt signaling pathways, AMPK signaling pathway, VEGF signaling pathway, and so on (Figure 4c).

## 4 DISCUSSION

These studies examined the blood pressure levels and prevalence of hypertension in current young athletes and former athletes, respectively. Among the young participants, mixed and sprint/power athletes showed higher SBP, and all types of athletes showed higher PP than non‐athlete controls. In contrast, the prevalence of hypertension was significantly lower in all types of former athletes than in the general population, regardless of current exercise habits. DNA methylation analyses suggested that aerobic and resistance exercise training commonly modified over 3000 DNA methylation sites and these were associated with several cardiovascular function‐related pathways. These results propose that exercise training, particularly exercise training including resistance exercise, may increase blood pressure at a young age, but this increased blood pressure may be an adaptation to the exercise training and is not associated with future risk of hypertension. Furthermore, previous exercise training experience at a young age decreases the risk of future hypertension by modifying DNA methylation, regardless of the sports discipline.

Although current athletes exhibit elevated blood pressure, this may be an adaptation to exercise training and does not influence the development of hypertension after retirement. It has been suggested that resistance and endurance training‐induced increased PP is an adaptation to the exercise training due to an increased cardiac function (Yoshioka et al., 2021), and the cardiovascular adaptation to the exercise training returns to the baseline levels after the detraining period (Miyachi et al., 2004; Mustata et al., 2004). In the present study, although current athletes showed higher blood pressure than the non‐athlete controls, former athletes did not exhibit a higher prevalence of hypertension than the general population. Because most of the former athletes in the present study were not professional and discontinued training after graduation from college, the detraining period was suggested to be sufficient to normalize the blood pressure. Taken together, these results suggest that exercise training‐induced cardiovascular adaptation at a young age is temporary and does not increase future CVD risk.

Both endurance and sprint/power former athletes exhibited lower prevalences of hypertension than the general population in the present study. Because protective effects of aerobic exercise against CVD are well‐established (Matsubara et al., 2014; Tanahashi et al., 2014; Tanaka et al., 2000), it was reasonable that former endurance athletes showed a lower prevalence of hypertension than the general population. Although it has been suggested that resistance training increases blood pressure (Miyachi et al., 2004; Otsuki et al., 2007), a large prospective study demonstrated that high muscular strength is protective against the incidence of CVD and cardiovascular death during the follow‐up period (Leong et al., 2015; Ortega et al., 2012). Because resistance‐trained people, including athletes, show higher muscle mass and strength than the non‐trained population, high muscular strength may be a possible mediator of our observation that former strength‐trained athletes also showed a lower prevalence of hypertension than the general population.

To explore the underlying mechanisms that both aerobic‐ and sprint/power‐trained former athletes showed lower prevalences of hypertension than the general population, we utilized publicly available DNA methylation datasets. These analyses revealed that the CpG sites commonly modified by both aerobic and resistance exercise training were associated with several cardiovascular function‐related pathways, including vascular smooth muscle contraction, apelin signaling pathway, and cholinergic synapse. Vascular dilation and contraction are regulated by vasoactive substances, such as nitric oxide (NO) and endothelin 1. Apelin is one of the regulators of NO (Ishida et al., 2004) and several studies demonstrated that apelin had vasodilation effects (Japp et al., 2008) and lowered arterial blood pressure (Tatemoto et al., 2001). Additionally, Fujie et al. (2014) suggested that exercise training‐induced increase in plasma apelin levels contributed to a decrease in arterial stiffness in middle‐aged and older adults. On the other hand, the autonomic nervous system, such as the sympathetic and parasympathetic nervous system, is also a strong regulator of blood pressure, and alternations in the sympathetic and parasympathetic nervous system contribute to the increase in blood pressure with aging (Baker et al., 2018; Tanaka et al., 2017). Additionally, other pathways, such as oxytocin signaling (Jankowski et al., 2020), cholinergic synapse (Buccafusco, 1996), TRP channels (Numata et al., 2016), HIF‐1α signaling (Huang et al., 2013), Notch signaling (Hofmann & Iruela‐Arispe, 2007), and sodium reabsorption (Chiolero et al., 2000), are also suggested to be associated with cardiovascular functions. Therefore, previous exercise training at a young age may influence the development of future hypertension through modifying the cardiovascular function‐related DNA methylation sites.

The Harvard Alumni Health Study, an epidemiologic study to examine the association between physical activity and health outcomes, suggested that current physical activity was the stronger determinant of current health status than past physical activities (Paffenbarger et al., 1986, 1993, 1994, 1997). Contrary, though, our study demonstrated that former college athletes showed a lower prevalence of hypertension than the general population. A possible explanation of these inconsistent observations is the volume of the past exercise training. While the highest category of physical activity in the Harvard Alumni Health Study was ≥3500 kcal per “week” (Paffenbarger et al., 1986), studies suggested that male athletes consumed around 4000 kcal per “day” (Frączek et al., 2019; Heydenreich et al., 2017). Participants of the present alumni study (i.e., Study 2) were former athletes, and their physical activity levels were speculated to be much higher than the general population and subjects in the Harvard Alumni Health Study. Altogether, the difference in the total volume of past exercise training may cause the different findings between the Harvard Alumni Health Study and the present study.

Our studies have several limitations. A lack of female subjects is a clear limitation of the present study. It is well known that the prevalence of hypertension increases after menopause in females. However, we did not have enough female former athletes to assess the prevalence of hypertension in the present study. Second, although we assessed current physical activity levels, we could not assess the details about the physical activity, aerobic and/or resistance exercise. Third, although we utilized the Japanese National Health and Nutrition Survey data as data on the prevalence of hypertension in the general population, this might have caused some biases because their lifestyles, such as exercise, dietary, or sleeping habits, might be different from those of former athletes. The last limitation is that we could not access the association between changes in DNA methylation and actual blood pressure. Although we analyzed the publicly available DNA methylation dataset (Lindholm et al., 2014; Seaborne et al., 2018), they did not have blood pressure data. These are the clear limitations of the present study, and future studies addressing these points are necessary.

In summary, although blood pressure levels were higher in young current athletes than in non‐athletes, the prevalence of hypertension in former athletes was lower than that in the general population. Additionally, aerobic and resistance exercise training commonly modified DNA methylation sites associated with several cardiovascular functions in the skeletal muscle. These findings suggest that high blood pressure in young athletes is an adaptation to exercise training and does not increase the risk of hypertension. Furthermore, exercise training experiences at a young age prevent the development of future hypertension by modifying DNA methylation. It is well known that current physical activity has an important role to prevent CVD. In addition to this, the present study suggests that past exercise training or a physically active lifestyle at a young age also contributes to lowering the future risk of CVD.

## AUTHOR CONTRIBUTIONS

Hiroshi Kumagai, Yuki Someya, Tetsuhiro Kidokoro, Masaki Yoshioka, Youngju Choi, Kaname Tagawa, Seiji Maeda, Yoshimitsu Kohmura, Koya Suzuki, Hisashi Naito, and Noriyuki Fuku designed research; Hiroshi Kumagai, Eri Miyamoto‐Mikami, Yuki Someya, Tetsuhiro Kidokoro, Masaki Yoshioka, Youngju Choi, Kaname Tagawa, Yoshimitsu Kohmura, Koya Suzuki, Shuichi Machida, and Noriyuki Fuku performed the experiments; Hiroshi Kumagai, Brendan Miller, Michi Emma Kumagai, and Masaki Yoshioka analyzed the data; Hiroshi Kumagai, Brendan Miller, and Michi Emma Kumagai prepared the figures; Hiroshi Kumagai, Eri Miyamoto‐Mikami, Brendan Miller, and Noriyuki Fuku drafted the manuscript; Hiroshi Kumagai and Masaki Yoshioka edited and revised the manuscript; all authors approved the final version of the manuscript.

## FUNDING INFORMATION

This research was supported in part by JSPS KAKENHI Scientific Research (B) (18H03155 to Noriyuki Fuku), Young Scientists (A) (17H04752 to Eri Miyamoto‐Mikami), Young Scientists (18 K17863 to Hiroshi Kumagai), and by the Institute of Health and Sports Science & Medicine, Juntendo University.

## CONFLICT OF INTEREST

The authors have no conflicts of interest directly relevant to the content of this article.

## ETHICS STATEMENT

The studies were approved by the Ethics Committees of Juntendo University and the University of Tsukuba. Written informed consent was obtained from each participant in accordance with the tenets of the Declaration of Helsinki.

## Supporting information




Figure S1
Figure S2
Table S1
