# Supplementary material for: Sports activities at a young age decrease hypertension risk—The J‐Fit + study
Source: Physiol Rep. 2022 Jun 27;10(12):e15364. doi: 10.14814/phy2.15364 (PMC9234749; doi:10.14814/phy2.15364)
Supplement: Supplementary file 1 — Figure S1 Figure S2 Table S1 [file PHY2-10-e15364-s001.pdf]

Sports activities at a young age decrease hypertension risk – the J-Fit$^+$ study

Hiroshi Kumagai[1,2], Eri Miyamoto-Mikami[1], Yuki Someya[1], Tetsuhiro Kidokoro[4], Brendan Miller[2], Michi Emma Kumagai[2,3], Masaki Yoshioka[5,6], Youngju Choi[7], Kaname Tagawa[5], Seiji Maeda[8], Yoshimitsu Kohmura[1], Koya Suzuki[1], Shuichi Machida[1], Hisashi Naito[1], Noriyuki Fuku[1]

[1] Graduate School of Health and Sports Science, Juntendo University, Chiba, Japan
[2] The Leonard Davis School of Gerontology, University of Southern California, California, USA
[3] Department of Psychiatry, David Geffen School of Medicine, University of California, Los Angeles, California, USA
[4] Faculty of Sport Science, Nippon Sport Science University, Tokyo, Japan
[5] Graduate School of Comprehensive Human Sciences, University of Tsukuba, Japan
[6] Japan Society for the Promotion of Science, Chiyoda-ku, Tokyo, Japan
[7] Institute of Sports & Arts Convergence, Inha University, Korea
[8] Faculty of Sport Sciences, Waseda University, Saitama, Japan

Running head: Past sports activities and hypertension

Correspondence:
Hiroshi Kumagai, Ph.D.
The Leonard Davis School of Gerontology
University of Southern California, California
3715 McClintock Ave, Los Angeles, CA 90089, USA.
Tel: +1 213-821-6073
E-mail: kumazin7@gmail.com

and

Noriyuki Fuku, Ph.D.
Graduate School of Health and Sports Science
Juntendo University
1-1 Hiraka-gakuendai, Inzai-city, Chiba 270-1695, Japan.
Tel: +81 476-98-1001
Fax: +81 476-98-1011
E-mail: noriyuki.fuku@nifty.com

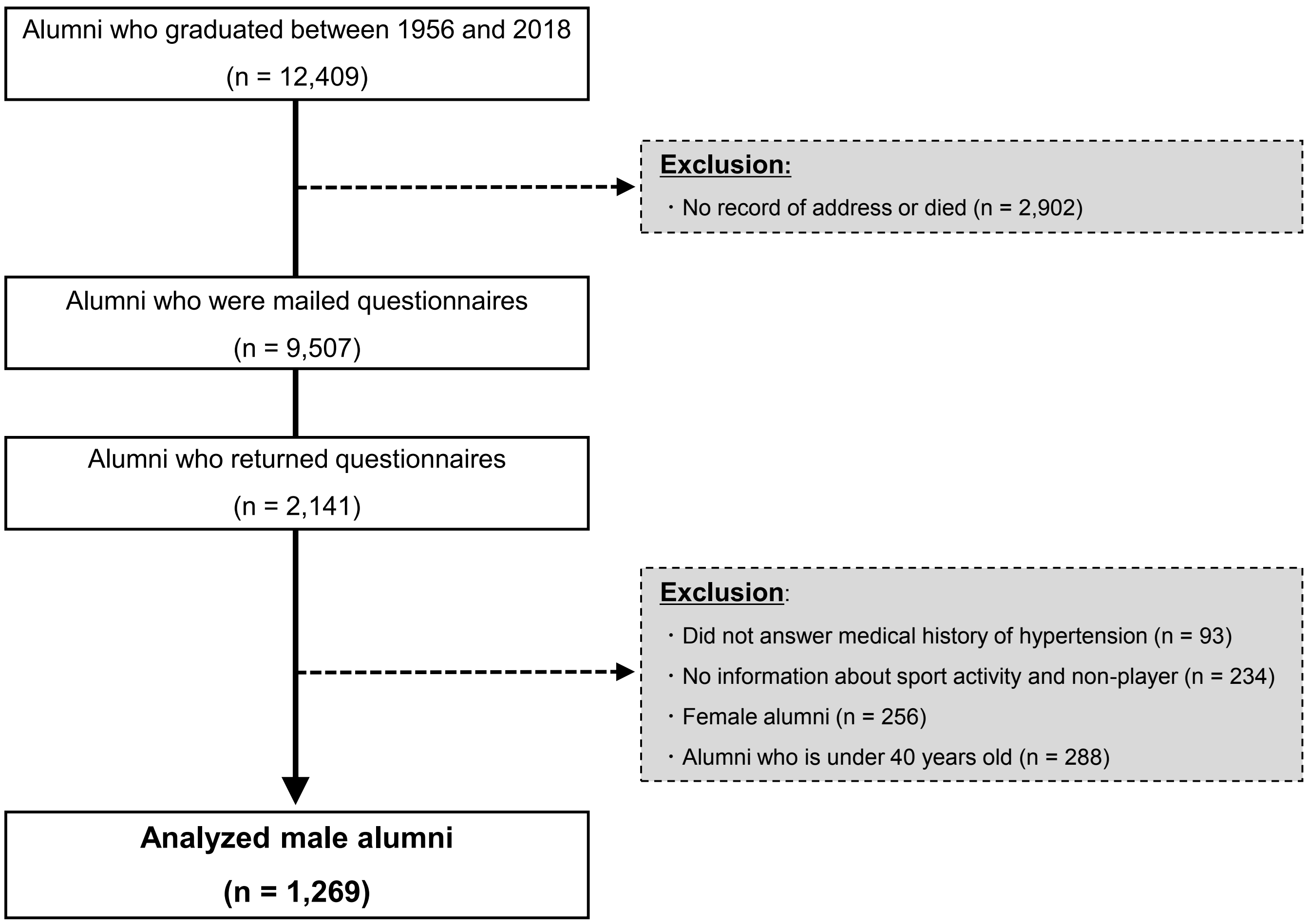

Supplementary Figure 1. A flowchart of participant inclusion among former athletes.

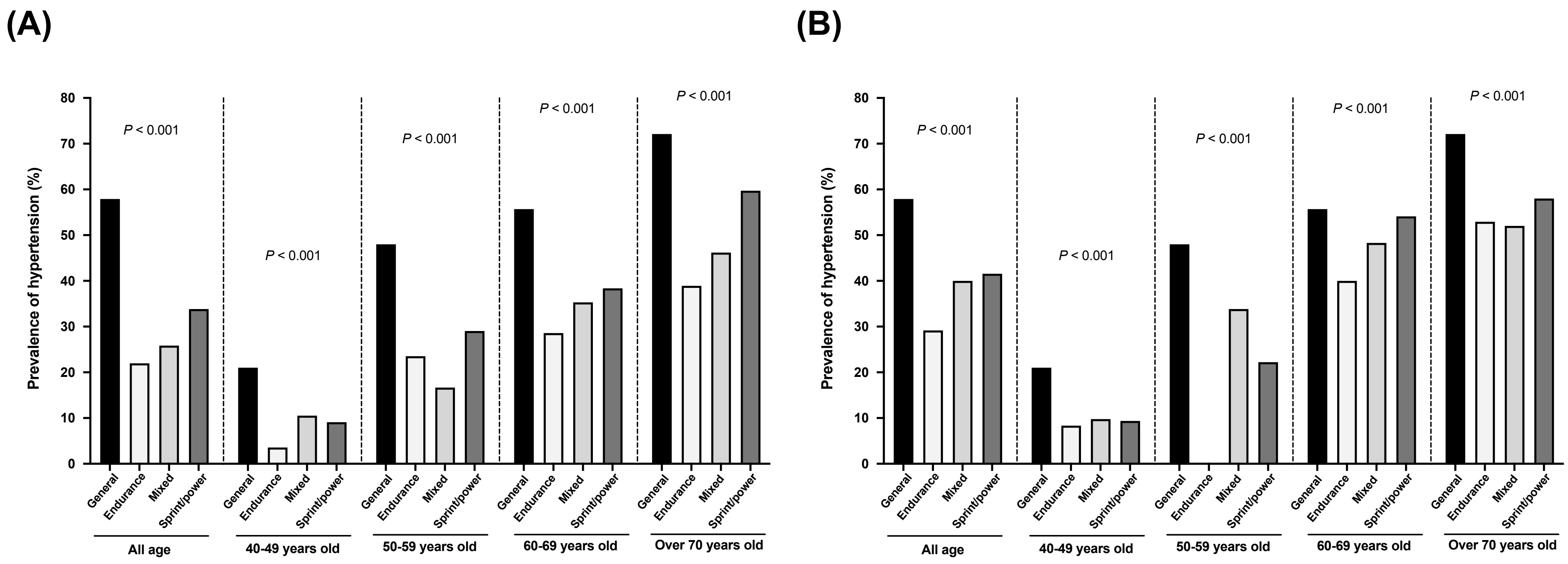

Supplementary Figure 2. Prevalence of hypertension in physically active (**A**) and physically inactive former athletes (**B**). Physical activity level was not considered in the general population. People who use antihypertensive medication were defined as hypertension in both former athletes and the general population.

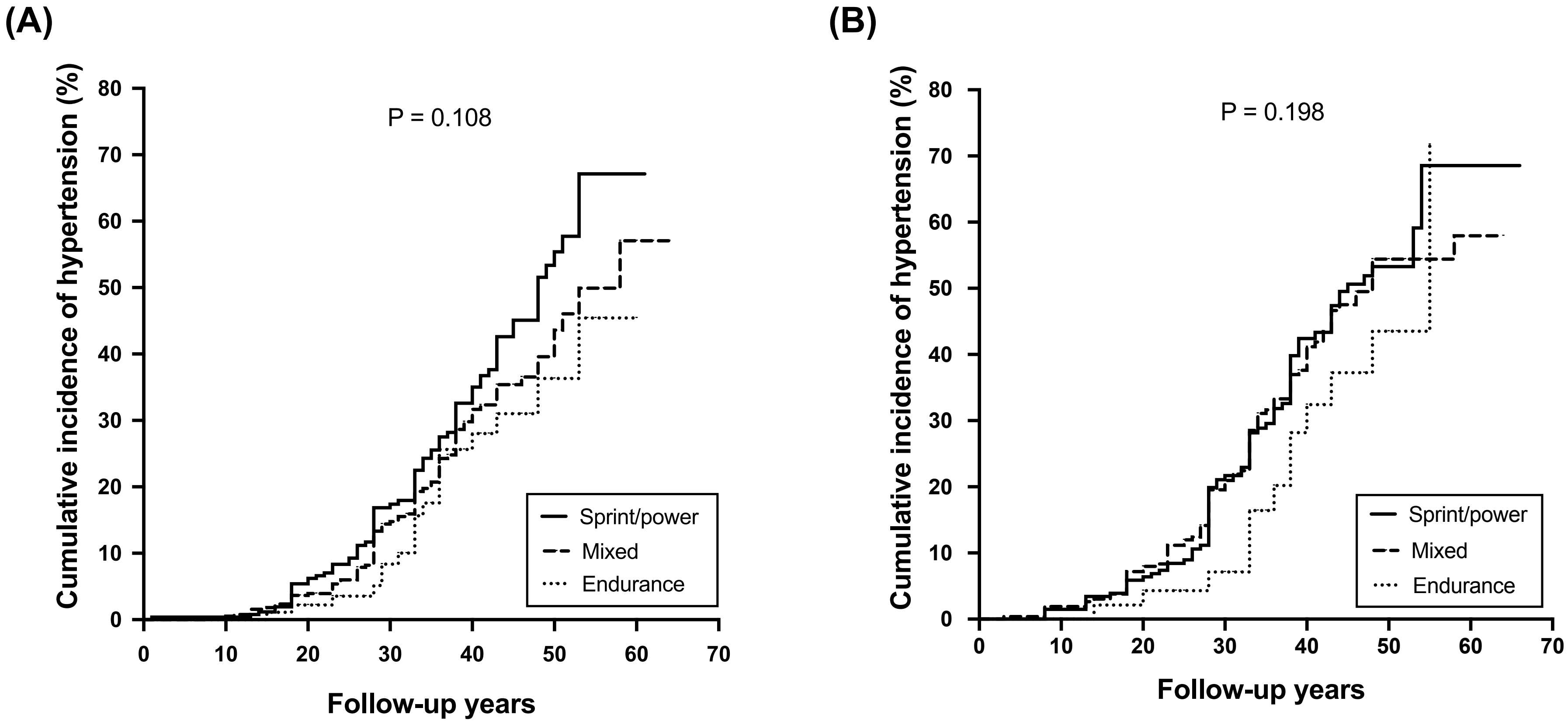

Supplementary Figure 3. Cumulative incidence curve for hypertension during the follow-up period in former athletes with current physical activity (**A**) and without current physical activity (**B**). Former athletes who use antihypertensive medication were defined as hypertension.

Supplementary Table 1. The adjusted hazard ratio for hypertension

	Hazard ratio	95% CI	P-value
Age, years	0.99	0.98-1.01	0.290
BMI, kg/m^2	1.16	1.12-1.22	< 0.001
Physical activity, yes/no	0.80	0.65-0.98	0.029
Smoking, yes/no	0.89	0.65-1.18	0.432
Drinking, yes/no	1.28	0.99-1.66	0.054
Sport type			
Endurance (reference)	-	-	-
Mixed	1.24	0.87-1.84	0.246
Sprint/power	1.50	1.04-2.23	0.029

CI: confidence interval.
